# Mindfulness as a protective factor against online risk-taking in middle school-aged children: the predictive effects of demographic and personal characteristics

**DOI:** 10.3389/fpsyg.2026.1676671

**Published:** 2026-02-04

**Authors:** Rabia Şengün Afşin, Özcan Doğan, Ayşe Koçyiğit Özlü

**Affiliations:** 1 Child Care and Youth Services Department, Buharkent Vocational School, Aydin Adnan Menderes University, Aydın, Türkiye; 2 Child Development Department, Faculty of Health Sciences, Hacettepe University, Ankara, Türkiye

**Keywords:** adolescence, children, mindfulness, online risk-taking, middle school

## Abstract

Online risk-taking among middle school-aged children is a growing concern, along with its potential consequences. With the widespread use of internet technologies, the likelihood of children encountering online risks can increase in parallel with the increase in usage. To date, there has been very little research on factors that could protect against online risks. The current study aims to fill this knowledge gap by measuring online risk-taking and mindfulness levels in a sample of 574 middle school students in Ankara, Türkiye, using descriptive and correlational screening models to determine the relationship between mindfulness and online risk-taking among middle school-age children and how demographic and personal characteristics predict both variables. Data were collected using the “Online Risk-Taking Scale” to measure online risk-taking levels, the “Mindfulness Scale for Children and Adolescents” to measure mindfulness attitudes, and the “Demographic and Personal Information Questionnaire” to measure demographic and personal characteristics. The results indicate a significant negative correlation between mindfulness and online risk-taking; demographic and personal characteristics such as age, gender, academic achievement, and daily screen time significantly influence online risk-taking; age, gender, birth order, father’s employment status, academic achievement, social relationships, and daily screen time significantly influence mindfulness. These findings emphasize the importance of mindfulness in online risk-taking, highlight the need for further research on this topic in this age group and on mechanisms to prevent children from engaging in risky behavior online; underscore the need for collaboration among all environmental systems in which children are embedded to keep them safe online; and stress the importance of developing educational programs and interventions to enhance children’s mindfulness.

## Highlights

- Developing mindfulness skills in middle school-aged children can reduce online risk-taking behaviors.
- Age, gender, academic achievement, and daily screen time significantly influence online risk-taking; age, gender, birth order, father’s employment status, academic achievement, social relationships, and daily screen time significantly influence mindfulness. These factors should be considered when planning interventions.
- Interventions aimed at reducing online risk-taking and raising mindfulness could focus specifically on adolescents, boys, children with low academic performance, and those with excessive screen time.
- School-based intervention programs that include mindfulness training can be integrated into middle school curricula to promote safe online behaviors.
- Effective prevention requires collaboration among parents, teachers, and school counselors to create supportive environments that help students maintain mindfulness and make safer online choices.

## 1 Introduction

Digital technology has brought about many fundamental changes in all areas of life in the 21st century and continues to do so (Burns et al., 2019). In particular, online usage, especially with internet technologies, has brought about numerous benefits that make life easier, such as developing the social and technical skills necessary for success today, getting to know people from different cultures and countries, learning new things, discovering areas of interest, doing things quickly and easily, and the ability to shop from the comfort of one’s home. This rapid development, change, and spread, with its countless benefits that make life easier, can lead to a series of changes not only in the lives of adults but also in the lives of children. With this transformation, online use offers opportunities to humanity with its positive benefits that make life easier, but at the same time, it also harbors new areas of risk with its negative effects on health, economics, psychology, and other disciplines, especially for children (Abaido, 2020; Faraz et al., 2022; Livingstone et al., 2017; Livingstone and Stoilova, 2019; Wang et al., 2025).

The widespread use of digital technologies deeply affects children’s social, emotional, and cognitive development (Livingstone et al., 2017). Understanding the profound impact of the internet on children is possible through a balanced assessment of the positive and negative effects of online interactions. Recent studies show a striking increase in the rate of internet technology use among children and adolescents (Livingstone and Helsper, 2007; Tüik, 2024). Compared to adults, adolescents use technological devices such as computers, smartphones, and tablets more frequently. According to United Nations estimates, 77% of children aged 15–24 will be using the internet in 2023; in many countries, the internet access rate among adolescents exceeds 90%; and 91% of adolescents in the United States use the internet daily (Gross, 2004). According to the Turkish Statistical Institute’s “Research on the use of information technologies among children,” the internet usage rate among children aged 6–15 was 82.7% in 2021 and rose to 91.3% in 2024 (Tüik, 2024). These data reveal the increasingly central role of digital technologies in children’s lives. Despite this increase in online usage, services and products to protect children are not taking sufficient steps, and as a result of these omissions, children may be disadvantaged in online environments (Jang and Ko, 2023).

Children’s online use is constantly changing with innovative applications, games, and immersive social media applications, and the increase in use means that the experiences children may encounter in these areas and the potential risks associated with them remain largely unexplored and insufficiently understood (Maheux et al., 2024; Noll et al., 2022). Children’s online use can include activities such as messaging, emailing, shopping, gaming, information gathering, education, browsing social networking sites, and so on. According to a study, more than half of children and all adolescents have accounts on social networking sites, and children use these accounts to disclose information about themselves, share personal information such as photos, and chat with strangers (Beebe et al., 2004). A study by the Turkish Statistical Institute shows that children use the internet for watching videos, participating in online classes, doing homework or learning, playing games and downloading games, social media, listening to or downloading music, and messaging (Tüik, 2024). Internet use may also lead to exposure to risks present in online environments. Indeed, it is noted that online risk behaviors have become more widespread due to the popularity of the internet and represent a significant health issue (Livingstone and Haddon, 2012). As children spend more time online, they are exposed to online risks such as sharing personal information, exposure to inappropriate content, and cyberbullying (Livingstone and Haddon, 2012). Exposure to these situations raises concerns about children’s health, development, and wellbeing. Research has shown that communicating with strangers online, spreading fake news, not paying attention to personal privacy, exposure to illegal content, shopping without parental knowledge, sharing photos, being exposed to bullying, bullying, and deceptive or fraudulent applications, among others, can occur; and this exposure is claimed to have negative effects on children’s physical health, mental health, social development and academic achievement; to increase the likelihood of children developing self-harming or suicidal thoughts or actions; and to lead to a loss of control by creating negative effects on mental and physical health (Arokiasamy et al., 2019; Caplan, 2010; Çelen et al., 2011; Gamez-Guadix et al., 2012; Jang and Ko, 2023; Kanan et al., 2018; Livingstone and Helsper, 2007; Lo et al., 2020; Wang et al., 2025). These situations cause problems, particularly placing children in a disadvantaged group and making the internet environment risky for children (Stamoulis, 2009). For all these reasons, developing protective measures to safeguard children against online risks is of great importance.

In studies conducted in the Turkish context, it is recommended to increase the mindfulness of families and teachers on this issue, create a safe internet environment, and use prevention programs to protect children from online risks; parents should be able to guide their children by developing their internet usage skills, monitoring and reducing the time children spend online, preventing the sharing of personal information on social media through parental control, adjusting privacy settings, maintaining good communication with children, informing them in advance about online risks, making them feel that they have a haven to turn to in case of danger, and supporting children’s digital literacy skills from an early age through mindfulness-raising activities; it is argued that policies to protect children from online risks should be developed and mindfulness-raising activities for children should be carried out (Çelen et al., 2011; Çıkman et al., 2017; Özkaya, 2023). The Organization for Economic Co-operation and Development (OECD) offers recommendations on principles, comprehensive policies, international cooperation, and internet service providers for the use of safe and beneficial digital environments for children (OECD, 2021). Ultimately, studies conducted worldwide and in Türkiye emphasize that online experiences can be positive and contribute to children’s development while reducing risks (Çelen et al., 2011; OECD, 2021). Despite a growing number of studies on this topic, empirical investigations focusing on the psychological, demographic, and personal characteristics of online risk-taking are scarce. This study first examines the relationship between online risk-taking and mindfulness and then investigates whether demographic and personal characteristics are predictive factors. Finally, recommendations are made on this topic to all environmental systems that affect children.

### 1.1 Children and online risks

Online environments contain both useful and harmful content. These harmful elements are referred to as online risks. Violence, fake news, racism, pornography, cyberbullying, sexual abuse, violation of personal privacy, advertisements, games, communication with strangers, content inappropriate for children’s age, and digital addiction are some of these risks. Livingstone and Stoilova (2021) classify online risks for children as content risks, communication risks, conduct risks, and contract risks (4C: Content, Contact, Conduct, Contract), providing a comprehensive framework (Livingstone and Stoilova, 2021). All these risks can negatively affect the quality of life of children and adolescents (Gonzalez- Cabrera et al., 2018).

Research shows that 49% of children communicate with strangers online; incidents of online abuse against children are increasing (Wolak et al., 2008; Wolak et al., 2010); across Europe, up to 91% of young people share their personal information online, four out of ten view pornography, approximately one-third are exposed to violent or hateful content, nearly one-fifth experience bullying, one in three receive unwanted sexual comments, and one in five meet someone they met online in person (Staksrud and Livingstone, 2009). 58% of the risks children encounter online are content risks, 19% are behavioral risks, 13% are communication risks, and 10% are other risks (Livingstone et al., 2014). Screen time in primary school students, along with parental supervision, is the main determinant of children’s involvement in cyberbullying and online harassment (Tintori et al., 2023).

Online risks can have various effects on children. Exposure to inappropriate content can negatively affect children’s behavior and values. Negative sharing can affect children’s online reputation and influence others’ thoughts and behavior toward them.

Research has linked online risks to psychological problems such as low self-esteem, depression, anxiety, and social isolation, an increase in violent behavior, the normalization of inappropriate content, behavioral changes in children, and an increase in suicidal thoughts (Hinduja and Patchin, 2010); excessive internet use can cause addictive behavior in children, which can lead to academic failure, depression, social isolation, anxiety, low self-esteem, and physical, mental, and emotional health problems (Kuss and Griffiths, 2012); internet addiction can cause a decline in individuals’ quality of life (Weinstein and Lejoyeux, 2010); smartphone addiction in young children reduces right brain function (Rajeswari, 2022); reducing the time spent on devices can reduce exposure to online risks (Jang and Ko, 2023); misleading information on the internet can negatively affect children’s worldviews because they do not know how to distinguish between true and false news (McGrew et al., 2018); children may be more vulnerable to misinformation than adults due to their insufficient digital media literacy skills, and their lack of critical thinking skills to identify harmful situations in online environments can make them vulnerable consumers and lead them to make wrong decisions shows (Jang and Ko, 2023; Kahne and Bowyer, 2018).

Given these results, the official recognition of internet access as a fundamental human right (La Rue, 2011) and the reality that children’s interaction in online environments cannot be prevented are of critical importance in understanding the psychological, demographic, and personal characteristics that can protect them from online risks and reduce their exposure to such risks. A study found that the conscious use of technology is critical for university students to minimize the potential risks of online environments while maximizing their benefits (Bayanov et al., 2019). This highlights the urgent need for research into the effectiveness of mindfulness in reducing online risks and into preventive and predictive factors for online risks, with a particular focus on children. Furthermore, considering the age at which children encounter online risks, the positive effects of early interventions on children’s health and wellbeing must be considered. Collecting reliable data to understand this phenomenon more deeply can facilitate the creation and implementation of prevention programs aimed at reducing online risks and increasing mindfulness.

### 1.2 Mindfulness as a potential buffer

The topic of mindfulness among children is a relatively new area of research. Mindfulness, defined as the ability to be aware of one’s thoughts, feelings, and actions, has been embraced by Western popular culture in recent years due to its positive effects on mental health (Brown and Ryan, 2003). It has become the focus of interdisciplinary research in the 21st century and is increasingly becoming a subject of interest for those working in the fields of public health, psychology, neuroscience, and psychiatry (Braver and Lazar, 2025). The concept of mindfulness has its roots in Buddhism and is defined as the ability to focus one’s attention and mindfulness on the present moment—observing and experiencing thoughts, emotions, and physical states as they arise in each moment in a purposeful, receptive manner without judgment (Eberth and Sedlmeier, 2012; Mettler et al., 2023).

Mindfulness has been found to have a positive relationship with quality of life and wellbeing (Siebelink et al., 2019); mindful individuals are less angry, hostile, and emotionally unstable (Garofalo et al., 2020; Heppner et al., 2008); mindfulness is reliably negatively associated with many antisocial behaviors and online risky behaviors, including physical aggression, verbal aggression, and cyberbullying (Garofalo et al., 2020; Peters et al., 2015; Yuan et al., 2020); there is a negative association between mindfulness and online risky behaviors such as exposure to cyberbullying incidents, problematic gaming, problematic smartphone use, and problematic internet use (Calvete et al., 2017; Elhai et al., 2018; Emirtekin et al., 2020; Gámez-Guadix and Calvete, 2016; Kircaburun et al., 2019; Royuela-Colomer et al., 2018); similarly, mindfulness may play a positive role in reducing online antisocial behaviors such as cyberbullying (Emirtekin et al., 2020; Yuan et al., 2020). According to various conceptual approaches, mindfulness develops self-regulation—defined as the ability to manage one’s emotions, thoughts, and behaviors—and increases positive social behaviors in individuals (Holzel et al., 2011; Perry-Parrish et al., 2016; Vago and Silbersweig, 2012); and it is associated with various positive outcomes such as reduced impulsivity and improved decision-making ability (Carmona i Farres et al., 2019).

Previous studies have shown that mindfulness is associated with various positive outcomes, such as reduced impulsivity and improved decision-making skills. Mindfulness-based interventions in school settings have been shown to positively affect psychological variables such as cognitive domain, stress, coping, and resilience (Carmona i Farres et al., 2019; Zenner et al., 2014). Therefore, it is assumed that children with higher levels of mindfulness may be less likely to take online risks. However, there are only a limited number of studies focusing on this topic. Additionally, the extent to which demographic and personal characteristics influence this relationship has not been sufficiently investigated.

While many studies highlight the protective role of mindfulness, some studies have shown weaker or non-significant results. For instance, one study found that the relationship between mindfulness and health was weak or indirect in certain internet-risk classes (Calvete et al., 2020). Similarly, a meta-analysis indicated that the association between mindfulness and problematic internet use was very weak or even non-significant in some studies (Fendel et al., 2025). In adolescents, mindfulness alone did not significantly predict aggression or risky behavior; rather, its influence depended on intervening variables (Hu et al., 2024). Furthermore, mindfulness programs implemented with at-risk youth showed no significant effects on some outcome variables (Rawlett and Scrandis, 2016). These discrepancies may be attributable to cultural differences, developmental stages, or the measurement tools employed. Such inconsistencies highlight the importance of further examining mindfulness and online risk-taking in different contexts, particularly among children.

Despite these mixed findings, overall evidence suggests that mindfulness has the potential to serve as a protective factor for children in online environments. Therefore, in this study, we hypothesize that higher levels of mindfulness may reduce children’s likelihood of engaging in online risk-taking behaviors, while acknowledging that this relationship may vary depending on developmental and contextual factors.

All these results suggest that individuals with high levels of mindfulness will approach all conditions in online environments with caution, and therefore their likelihood of engaging in risky behaviors in online environments may be lower. Individuals with high levels of mindfulness are expected to be aware of risky behaviors in online environments, such as sharing personal confidential information with strangers, and are aware of risky actions in online environments. It is expected that individuals with high levels of mindfulness will avoid risky behaviors in online environments.

The technological age has changed and transformed children’s social interaction, communication, and learning styles. However, the opportunities provided by online environments also pose a number of risks for children. The increasing frequency of exposure to online risks has led to the idea that there may be a positive relationship between mindfulness and the ability to counteract risks. It is thought that individuals with high levels of mindfulness may behave more cautiously when exposed to online risks and that mindfulness may protect against online risk-taking behavior and influence behavior.

It is thought that certain demographic and personal characteristics, such as age and gender, may play a role in online risk-taking. For example, older children may be more fearless in the face of online risks due to the developmental characteristics of adolescence. It is also thought that the family’s monthly income may affect access to technology, and therefore access to online resources, which in turn may affect exposure to online risks. It is thought that good social relationships may reduce participation in online environments, while parents’ educational level may affect children’s mindfulness, thereby influencing online risk-taking.

Although there are existing studies on mindfulness and online risks (Calvete et al., 2020), there is a research gap regarding the predictive effect of study group differences and demographic-personal characteristics on online risks and mindfulness. This study aims to address this gap in the literature and provide valuable insights into how demographic and personal characteristics may predict mindfulness and online risk-taking among middle school students. By identifying these differences, educators and all environmental systems related to children can develop interventions aimed at raising mindfulness in children and encouraging behaviors that reduce online risks.

### 1.3 Current study

A key issue addressed in this research is the lack of empirical clarity regarding the extent to which mindfulness may serve as a protective factor against online risk-taking in middle school children. While mindfulness has been associated with reduced risky behavior in some adolescent and adult populations, little is known about whether similar patterns hold true for younger age groups and how demographic and personal variables may influence this association.

Despite growing interest in children’s online risk-taking behavior, there are few studies examining protective psychological factors (e.g., mindfulness) and demographic and personal characteristics that could protect middle school-aged children from online risky behavior. The current literature shows that mindfulness -based approaches for children and adolescents are still in their infancy but have yielded positive results in terms of applicability; mindfulness reduces adolescents’ exposure to antisocial content in the media, reduces online risky behavior through higher risk perception, and has a negative relationship with health-related quality of life associated with risky online behavior; conversely, mindfulness has a more positive relationship with health-related quality of life; mindfulness training for children has the potential to develop attention and concentration skills, memory, self-acceptance, self-management, and self-understanding skills (Burke, 2010; Calvete et al., 2020; Hooker and Fodor, 2008). However, the factors that may predict online risk-taking, particularly in childhood, have not been sufficiently researched. Furthermore, online risk-taking and mindfulness are thought to vary depending on specific demographic and personal characteristics, further complicating the behavioral landscape.

To address these gaps, this study investigates how online risk-taking behavior is related to mindfulness in a sample of middle school-aged children, particularly whether demographic and personal characteristics play a predictive role. The study focuses on two main objectives:

*Objective 1.* To quantitatively determine the relationship between online risk-taking and mindfulness in middle school-age children.

*Objective 2*. To identify demographic and personal variables that predict online risk-taking and mindfulness.

In line with these objectives, the following research questions guide the study:

- Is there a significant relationship between children’s mindfulness levels and their online risk-taking behaviors?
- Do children’s online risk-taking behaviors significantly differ according to demographic and personal variables?
- Do children’s mindfulness levels significantly differ based on demographic and personal variables?

By exploring these questions, the study aims to contribute to the limited but growing literature on mindfulness as a protective factor in digital contexts and to highlight the role of developmental and contextual variables in shaping children’s online experiences.

This study offers meaningful practical contributions beyond its theoretical contributions to the field. Defining mindfulness as a protective factor against online risk-taking among middle school children can contribute to the development of school-based intervention programs and digital safety education. School counselors and guidance teachers, child psychologists, and educators can integrate mindfulness practices into the curriculum to increase children’s self-control and awareness in online environments. Furthermore, understanding how demographic and personal factors affect these variables in middle school-aged children allows for the adaptation of preventive strategies to the specific needs of different student groups. All this information can guide policymakers and all public institutions working with children in designing programs that reduce online risk-taking during early adolescence, impart age-appropriate digital literacy skills, and develop self-control skills.

By explicitly addressing these gaps, the current study not only advances theoretical understanding but also provides evidence to inform the development of contextually sensitive, developmentally appropriate interventions that can enhance children’s resilience in online environments.

## 2 Materials and methods

### 2.1 Study design

Descriptive and correlational survey designs were employed in the present study. The use of a quantitative research method allowed for the collection and analysis of numerical data that could be generalized to a larger sample (Can, 2024). Through a descriptive approach, the levels of mindfulness and online risk-taking among middle school children were examined in greater depth.

The sample was selected using simple random sampling (Can, 2024). A list of all public middle schools in Çankaya District, Ankara, during the second semester of the 2023–2024 academic year was compiled and numbered (a complete list of these schools was obtained from the District Directorate of National Education), and five schools were randomly selected by drawing lots. Principals of the selected schools were contacted and invited to participate in the study; all five schools agreed to participate (school participation rate 100%).

### 2.2 Participants

In this cross-sectional study, the sample consisted of 574 students (54.0% girls; mean age = 12.75; SS = 1.33) enrolled in five public middle schools randomly selected from public middle schools in Çankaya District, Ankara, Türkiye, during the second semester of the 2023–2024 academic year.

To determine the minimum required sample size for detecting a statistically significant relationship between mindfulness and online risk-taking behavior, an a priori power analysis was conducted using the G*Power 3.1.9.7 software. A correlation analysis was selected as the statistical test, with a two-tailed test assuming a directional hypothesis.

The effect size was set to ρ = 0.15, representing a small to medium effect based on Cohen’s (1988) conventions (Cohen, 1988). The significance level (α) was fixed at .05, and the desired statistical power (1 – β) was set to .95, ensuring a 95% probability of correctly rejecting the null hypothesis when the effect exists. Under these parameters, the analysis indicated that a minimum total sample size of 567 participants would be required to achieve the desired power. To prevent data loss, data collection was carried out so as to exceed the minimum sample size.

Information on the demographic and personal characteristics of the participants is presented in Table 1.

**TABLE 1:** Demographic and personal characteristics of the participants (*N* = 574).

(*N* = 574)	Number	Percentage %
**Age of children (Average ± SS = 12,75 ± 1,33)**		
10–12 years old	251	43.7
13–18 years old	323	56.3
**Gender of children**		
Girl	310	54.0
Boy	264	46.0
**Mother’s education level**		
Did not go to school	6	1.0
Primary school dropout	5	0.9
Primary school	35	6.1
Middle school	38	6.6
High school	141	24.6
University	349	60.8
**Father’s education level**		
Did not go to school	1	0.2
Primary school dropout	9	1.6
Primary school	22	3.8
Middle school	24	4.2
High school	115	20.0
University	403	70.2
**Number of siblings of children (including self)**		
1	108	18.8
2	252	43.9
3	159	27.7
4 and above	55	9.6
**Birth order**		
1	283	49.3
2	204	35.5
3	70	12.2
4 and above	17	3.0
**Family monthly income**		
0–20 thousand Turkish lira	92	16.0
20–40 thousand Turkish lira	164	28.6
40–60 thousand Turkish lira	154	26.8
60 thousand Turkish lira and above	164	28.6
**Mother’s employment status**		
Working	551	96.0
Not working	23	4.0
Father’s employment status		
Working	268	46.7
Not working	306	53.3
**Children’s academic achievement**		
Good	358	62.4
Middle	206	35.9
Bad	10	1.7
**Children’s social relationships with their friends**		
Good	431	75.1
Middle	130	22.6
Bad	13	2.3
**Daily television-tablet-phone-computer usage time of children**		
0–2 H	260	45.3
2–4 H	220	38.3
4–6 H	69	12.0
6 H or more	25	4.4

According to Table 1, 43.7% of the participants were 10–12 years old, and 56.3% were 13–18 years old. The mean age was 12.75 ± 1.33 years. Of the participants, 54.0% were girls. Regarding parental education levels, 60.8% of mothers and 70.2% of fathers had a university degree. Regarding family structure, 43.9% of the participants had two siblings (including themselves), and 49.3% were the first child. In terms of monthly family income, 16.0% reported 0–20,000 TL, 28.6% reported 20,000–40,000 TL, 26.8% reported 40,000–60,000 TL, and 28.6% reported over 60,000 TL. Among mothers, 4.0% were not employed, while 46.7% of fathers were employed. Of the students, 62.4% reported good academic achievement, and 75.1% reported good social relationships with peers. Daily screen time (television, tablet, phone, or computer use) was between 0 and 2 h for 45.3% of participants.

### 2.3 Data collection tools

#### 2.3.1 Demographic and personal information questionnaire

Participants’ demographic and personal characteristics were collected using a researcher-developed questionnaire designed specifically for this study. This structured form aimed to gather comprehensive background information to support analyses of potential predictors of online risk-taking and mindfulness.

The questionnaire included items on the following variables:

- Age and gender of the participant
- Parental education level (mother and father separately)
- Number of siblings and birth order
- Monthly family income
- Parental employment status
- Perceived academic achievement
- Quality of peer social relationships
- Daily screen time, including time spent on devices such as televisions, tablets, smartphones, and computers

All items were constructed to be age-appropriate and easily understood by middle school-aged children. This form provided the demographic foundation for subsequent analyses examining group differences and predictive relationships.

#### 2.3.2 Online risk-taking scale

The Online Risk-Taking Scale, developed by Afşin et al. (2024), was used to assess the frequency and nature of online risk-taking behaviors among preadolescents. The scale consists of 14 items; each reflecting behaviors engaged in over the previous 6 months. It captures a range of risky online activities, including, but not limited to, sharing personal identity information and arranging face-to-face meetings with individuals encountered online.

The instrument is a 5-point Likert-type scale, with response options ranging from 1 (*Not true*) to 5 (*Always true*). Total scores can range from 14 to 70, with higher scores indicating a greater propensity for engaging in online risk-taking behaviors. A score of 42 serves as the interpretive midpoint, with scores above this threshold suggesting more frequent engagement in risky digital activities.

Psychometric analysis indicates that the scale demonstrates excellent reliability, with a Cronbach’s alpha of .922 and McDonald’s omega of .932. Exploratory factor analysis (EFA) established construct validity, yielding a Kaiser-Meyer-Olkin (KMO) value of .927 and a significant Bartlett’s test of sphericity [χ^2^(91) = 1883.213, *p* < 0.01], confirming the appropriateness of the factor structure. The scale accounted for 53.40% of the total variance, exceeding the commonly accepted 50% benchmark for robust construct validity (Afşin et al., 2024).

#### 2.3.3 Mindfulness scale for children and adolescents

The Mindfulness Scale for Children and Adolescents was originally developed by Greco et al. (2011) to assess mindfulness skills in individuals aged 9 years and older (Greco et al., 2011). Recognizing the scarcity of developmentally appropriate instruments for younger populations, this scale was designed to measure mindfulness through age-appropriate language and structure. The original version comprises 10 negatively worded items, all of which are reverse-coded. Responses are rated on a 5-point Likert scale, ranging from 1 (*Not true*) to 5 (*Always true*), with higher scores indicating greater levels of mindfulness.

The original scale demonstrated good psychometric properties, with a Cronbach’s alpha of .81. Unlike many adult mindfulness instruments that adopt multi-dimensional structures, this scale reflects a single-factor model, which aligns with the developmental limitations in children’s ability to self-regulate and introspect.

The Turkish adaptation of the scale was conducted by Çıkrıkçı (2016) and validated on a sample of 660 students from Grades 5 through 11. Initial confirmatory factor analysis (CFA) revealed that the single-factor structure of the original scale did not fully replicate in the Turkish context. As a result of item analysis, two items were removed, and a revised single-factor structure was established. The Turkish version demonstrated adequate internal consistency, with a Cronbach’s alpha of 0.73. The adaptation was carried out with permission from the original authors, ensuring the scale’s cultural and contextual validity.

This adapted version provides a valid and reliable tool for evaluating mindfulness in Turkish children and adolescents and is suitable for both research and educational applications (Çıkrıkçı, 2016). The Turkish short form comprises 8 items scored on a 5-point Likert scale from 0 (“not true”) to 4 (“always true”), yielding a total score range of 0–32, with higher scores indicating greater mindfulness.

### 2.4 Data collection process

**FIGURE 1:**
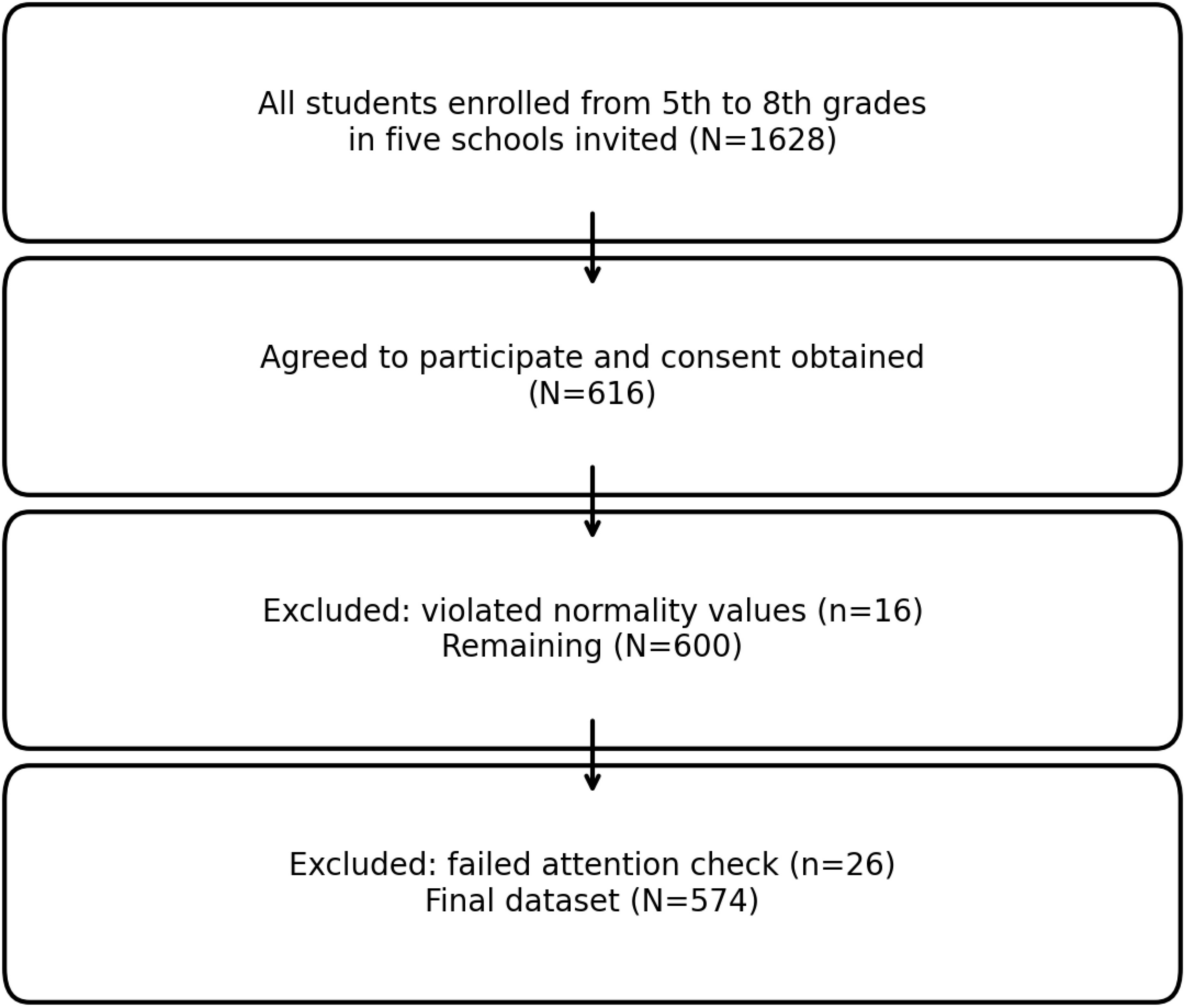
Participant flow chart.

All students enrolled from 5th to 8th grades in the five selected schools (1,628 students) were invited to participate in the study. Information about the study and links to the online survey (generated via Google Form) were distributed to parents via WhatsApp by school administrators to maximize participation. A total of 616 students (37.8%) provided parental and child consent and completed the survey. Data from 16 participants were excluded due to extreme outlier values that violated normality assumptions, and an additional 26 students were excluded for failing to respond correctly to an embedded attention-check item (e.g., “If you have read the previous section, please tick this box”). The final analytic sample therefore consisted of 574 participants. The participant flow is illustrated in Figure 1.

The study has the necessary ethics committee approval and permissions from relevant institutions. This information is included on the title page. All stages of the research complied with the ethical standards specified in the 1964 Declaration of Helsinki and its subsequent amendments. Data were collected between January 12 and April 4, 2024, through an online Google Form that included an information sheet and a consent form. To ensure confidentiality and anonymity, participants were asked not to share any personal information. Participants were also informed that they could withdraw from the study at any stage, ensuring flexibility and autonomy. Only those who voluntarily agreed after being informed were included in the study.

### 2.5 Data analysis

All statistical analyses in the current study were conducted in an exploratory manner to examine potential relationships and predictive contributions.

All statistical analyses were conducted using IBM SPSS Statistics version 26. The data were checked and cleaned before analysis. Normality of numerical variables was evaluated using skewness and kurtosis coefficients. The online risk-taking scale showed excessive kurtosis, and inspection indicated that 16 participants had extreme scores with z-values exceeding ± 1.96. This threshold was used to identify observations at the distributional tails that were also close to the scale floor, raising concerns about their influence on normality. These cases were excluded, and analyses were repeated with the remaining 574 participants. In this cleaned dataset, skewness and kurtosis values for all variables fell within the acceptable ± 2 range, suggesting that the assumption of normality was met. Therefore, parametric statistical methods were used in the study.

Descriptive analyses were performed to summarize the characteristics of the sample: frequencies and percentages were reported for categorical variables, while means and standard deviations were calculated for continuous variables.

To examine the relationships between continuous variables, Pearson’s correlation coefficient was employed. Group comparisons were conducted using appropriate parametric tests:

- Independent samples *t*-tests were used to assess differences between two independent groups.
- One-way analysis of variance (ANOVA) was used to compare more than two independent groups.
- When ANOVA results were statistically significant, the Tukey HSD *post hoc* test was applied to determine specific group differences.
- To assess the predictive effect of a single independent variable on a continuous dependent variable, simple linear regression analysis was performed.
- A hierarchical multiple regression analysis was conducted to examine the unique contribution of mindfulness to online risk-taking behavior. In the first step (Model 1), demographic and behavioral variables (age, gender, parental education, number of siblings, birth order, monthly income, parental employment status, academic achievement, social relationships, and daily screen time) were entered as predictors. In the second step (Model 2), mindfulness was added to assess its incremental predictive value.

The rationale for using hierarchical models was to determine whether mindfulness explained additional variance in online risk-taking behavior beyond the effects of demographic and personal characteristics. By first accounting for background variables in Model 1 and then adding mindfulness in Model 2, this stepwise approach allowed for the assessment of the incremental predictive power of mindfulness while controlling for potential confounding effects.

All statistical tests were two-tailed, and the level of statistical significance was set at *p* < 0.05. Given the number of subgroup comparisons, no multiplicity adjustments were applied; *p*-values are interpreted in an exploratory spirit, with effect sizes emphasized for interpretation.

Additionally, the reliability and fit indices of the DFA for the Online Risk-Taking Scale and Mindfulness Scale was evaluated (Table 2).

**TABLE 2:** Reliabilities and fit indices of the DFA for the online risk-taking scale and mindfulness scale.

Fit indices	Online risk-taking scale	Mindfulness scale	Recommended cutoff values	Acceptable cutoff values
CMIN/DF	1.060	1.308	< 2	<5
CFI	0.991	0.996	> 0.95	>0.90
NFI	0.873	0.981	>0.95	>0.90
RMSEA	0.010	0.023	< 0.06	<0.10
GFI	0.983	0.991	> 0.95	>0.90
AGFI	0.972	0.980	> 0.95	>0.90
Cronbach’s alpha (α)	0.872	0.812	≥ 0.70	≥ 0.70

According to the analysis, the CMIN/DF value was found to be 1.060 for the Online Risk-Taking Scale and 1.308 for the Mindfulness Scale. Both values are below the recommended cutoff of 2, indicating a good model fit. The CFI values were 0.991 and 0.996, respectively, both exceeding the 0.95 threshold, and the NFI values were 0.873 and 0.981. While the NFI value of the Online Risk-Taking Scale did not reach the recommended cutoff, the NFI value of the Mindfulness Scale was within the acceptable range (Baumgartner and Homburg, 1996; Marsh et al., 2006). Furthermore, the RMSEA values were 0.010 and 0.023, both of which indicate a good fit since they fall below the 0.06 cutoff (Byrne, 2001; Hu and Bentler, 1999). The GFI values were 0.983 and 0.991, both above the recommended threshold of 0.95 (Baumgartner and Homburg, 1996). Similarly, the AGFI values were 0.972 and 0.980, both exceeding the 0.95 cutoff (Schermelleh-Engel et al., 2003). Taken together, these findings show that the model fit indices of both scales demonstrate adequate construct validity. Internal consistency was confirmed using Cronbach’s alpha, with all scales demonstrating acceptable reliability (α > 0.70), supporting the suitability of the instruments for the study population.

## 3 Results

### 3.1 Common method bias

Given that the study relied on self-report measures, Harman’s single-factor test was used to assess the potential presence of common method bias (Podsakoff et al., 2003; Podsakoff et al., 2012). An exploratory factor analysis (EFA) was conducted on all items from the Online Risk-Taking Scale and the Mindfulness Scale for Children and Adolescents. The analysis revealed 8 factors with eigenvalues above 1, and the first factor accounted for 17.23% of the total variance, with an eigenvalue of 3.791. Since this is well below the commonly accepted threshold of 40%, it was determined that common method bias was not a concern in this study.

### 3.2 Results for descriptive statistics and correlation

The findings from the statistical analyses conducted on the collected data are presented below.

As shown in Table 3, the mean score on the Online Risk-Taking Scale was *M* = 16.39, *SD* = 2.89, while the mean score on the Mindfulness Scale for Children and Adolescents was *M* = 22.03, *SD* = 6.57.

**TABLE 3:** Descriptive statistics of variables.

Variables	Mean (M)	SD	Min	Max
Online risk taking scale	16.39	2.89	14	27
Mindfulness scale for children and adolescents	22.03	6.57	0	32

A Pearson correlation analysis (Table 4) revealed a moderate, statistically significant negative relationship between scores on the Online Risk-Taking Scale and the Mindfulness Scale for Children and Adolescents [*r* = –0.289, *p* < 0.05, CI (–0.362, –0.212)]. This finding suggests that higher levels of mindfulness are associated with lower levels of online risk-taking.

**TABLE 4:** Correlation of variables.

Variables	Mindfulness scale for children and adolescents	
Online risk taking scale	*r* (95% CI)	–0.289 (–0.362 / –0.212)
	*p*	0.000

*r*: Pearson Correlation Coefficient.

### 3.3 Results of ANOVA and *t*-test, regression, and hierarchical multiple regression

According to Table 5, significant group differences in online risk-taking and mindfulness scores were observed across several demographic and behavioral variables:

**TABLE 5:** Examination of the differences in online risk taking scale and mindfulness scale for children and adolescents scores according to demographic—personal characteristics.

Demographic and personal characteristics	Online risk taking scale	Mindfulness scale for children and adolescents
	Mean ± SD	Mean ± SD
**Age**		
10–12 years old	15.76 ± 2.41	22.71 ± 6.43
13–18 years old	16.88 ± 3.12	21.50 ± 6.64
*F*;*p*	–4.707;0.000*	2.199;0.028*
Effect size (Cohen’s *d*)	0.185	–0.396
**Gender**		
Girl	16.15 ± 2.81	21.34 ± 6.77
Boy	16.67 ± 2.96	22.84 ± 6.24
*t*;*p*	–2.138;0.033*	–2.739;0.006*
Effect size (Cohen’s *d*)	0.179	0.229
**Mother’s education status**		
Secondary school and below	16.36 ± 2.62	22.54 ± 6.62
High school	16.36 ± 3.38	22.03 ± 6.73
University	16.41 ± 2.73	21.91 ± 6.51
*F*;*p*	0.018;0.982	0.305;0.737
Effect size (η^2^)	0.000	0.001
**Father’s education status**		
Secondary school and below	15.77 ± 2.22	22.14 ± 6.42
High school	16.78 ± 3.18	22.09 ± 6.86
University	16.36 ± 2.87	22.00 ± 6.52
*F*;*p*	2.397;0.092	0.017;0.983
Effect size (η^2^)	0.008	0.000
**Number of siblings of children (including self)**		
1	16.04 ± 2.54	22.97 ± 5.83
2	16.25 ± 2.70	22.11 ± 6.67
3	16.60 ± 3.11	21.66 ± 6.93
4 and above	17.09 ± 3.52	20.89 ± 6.33
*F*;*p*	2.093;0.100	1.475;0.220
Effect size (η^2^)	0.011	0.008
**Birth order**		
1	16.31 ± 2.83	22.84 ± 6.18
2	16.45 ± 2.86	20.96 ± 6.93
3 and above	16.49 ± 3.14	21.92 ± 6.65
*F*;*p*	0.192;0.826	4.916;0.008*
Difference (Tukey)	–	1 > 2
Effect size (η^2^)	0.001	0.017
**Family monthly income**		
0–20 Thousand Turkish lira	16.32 ± 2.72	22.76 ± 7.31
Demographic and personal characteristics	Online risk taking scale	Mindfulness scale for children and adolescents
20–40 Thousand Turkish lira	16.67 ± 3.14	21.45 ± 6.43
40–60 Thousand Turkish lira	15.90 ± 2.83	22.03 ± 6.94
60 Thousand Turkish lira and above	16.61 ± 2.72	22.21 ± 5.89
*F*;*p*	2.376;0.069	0.843;0.471
Effect size (η^2^)	0.012	0.004
**Father’s employment status**		
Working	16.40 ± 2.90	21.93 ± 6.62
Not working	16.00 ± 2.41	24.39 ± 4.81
*t;p*	0.659;0.510	–2.359;0.026*
Effect size (Cohen’s *d*)	0.140	–0.375
**Mother’s employment status**		
Working	16.51 ± 28.2	21.95 ± 6.12
Not working	16.28 ± 2.94	22.10 ± 6.95
*t*;*p*	0.924; 0.356	–0.272; 0.785
Effect size (Cohen’s *d*)	0.077	–0.023
**Children’s academic achievement**		
Good	16.15 ± 2.72	22.95 ± 6.23
Medium/bad	16.79 ± 3.11	20.51 ± 6.86
*t*;*p*	–2.528; 0.012*	4.365; 0.000*
Effect size (Cohen’s *d*)	0.225	–0.376
**Children’s social relationships with their friends**		
Good	16.34 ± 2.97	23.08 ± 6.01
Medium/bad	16.54 ± 2.63	18.87 ± 7.17
*t*;*p*	–0.717; 0.474	6.312; 0.000*
Effect size (Cohen’s *d*)	–0.069	0.665
**Daily television-tablet-phone-computer usage time of children**		
0–2 h (1)	15.93 ± 2.58	23.14 ± 6.31
2–4 h (2)	16.65 ± 2.96	21.35 ± 6.51
4 h or more (3)	17.04 ± 3.31	20.56 ± 6.99
*F*;*p*	6.632; 0.001*	7.422; 0.001*
Difference (Tukey)	1 < 2.3	1 > 2.3
Effect size (η^2^)	0.023	0.025

*F*, One-Way Analysis of Variance (ANOVA); *t*, Independent Sample *t*-test.

*p < 0.05.

*Age*: Participants aged 13–18 show higher online risk-taking than those aged 10–12 (*p* = 0.000, Cohen’s *d* = 0.185). Younger participants (10–12 years) showed higher mindfulness (*p* = 0.028, Cohen’s *d* = –0.396).

- *Gender*: Boys scored higher in online risk-taking than girls (*p* = 0.033, Cohen’s *d* = 0.179). Boys also showed higher mindfulness scores (*p* = 0.006, Cohen’s *d* = 0.229).
- *Birth Order*: Mindfulness scores differed significantly across groups (*p* = 0.008, η^2^ = 0.017), with first-born children reporting higher mindfulness than second-born children.

*Father’s Employment*: Children whose fathers were not working reported higher mindfulness (*p* = 0.026, Cohen’s d = –0.375).

*Academic Achievement*: Children with good academic achievement scored lower in online risk-taking (*p* = 0.012, Cohen’s *d* = 0.225) and higher in mindfulness (*p* = 0.000, Cohen’s *d* = –0.376).

- *Daily Screen Time*: Participants with more than 2 h of screen use per day scored higher in online risk-taking (*p* = 0.001, η^2^ = 0.023), while those with < 2 h reported higher mindfulness (*p* = 0.001, η^2^ = 0.025).

As summarized in Table 6, a simple linear regression analysis was conducted to evaluate the predictive role of mindfulness on online risk-taking. The model was statistically significant (*F* = 52.144, *p* < 0.001) and did not exhibit autocorrelation issues (Durbin–Watson = 1.176). The model accounted for 8.2% of the variance in online risk-taking scores (Adjusted *R*^2^ = 0.082).

**TABLE 6:** Examination of the relationship between mindfulness scale scores and online risk-taking scale scores for children and adolescents.

Independent variables	β	Std. error	Std. β	*t*	*p*	95% CI for β (lower)	95% CI for β (upper)
Mindfulness	–0.127	0.018	–0.289	–7.221	0.000	–0.161	–0.092
Model statistics:	*F* = 52.144, *p* = 0.000*, *R*^2^ = 0.084, Adjusted *R*^2^ = 0.082, DW = 1.176						
**Model 1**							
Age	0.388	0.081	0.200	4.781	0.000	0.228	0.547
Gender	–0.476	0.236	–0.082	–2.015	0.044	–0.939	–0.012
Mother’s ed.	0.054	0.159	0.019	0.337	0.736	–0.259	0.366
Father’s ed.	0.373	0.173	0.115	2.152	0.032	0.033	0.713
Sibling no.	0.441	0.171	0.134	2.584	0.010	0.106	0.776
Birth order	–0.106	0.189	–0.029	–0.558	0.577	–0.478	0.266
Monthly inc.	–0.078	0.134	–0.028	–0.580	0.562	–0.342	0.186
Father’s emp.	–0.133	0.606	–0.009	–0.219	0.827	–1.323	1.058
Mother’s emp.	–0.313	0.263	–0.054	–1.188	0.235	–0.831	0.204
Academic ach.	0.531	0.240	0.096	2.210	0.028	0.059	1.004
Social rel.	–0.044	0.240	–0.008	–0.186	0.853	–0.515	0.426
Daily tv. etc. usage	0.377	0.148	0.108	2.553	0.011	0.087	0.667
Model statistics	*F* = 5.482, *p* = 0.000*, *R*^2^ = 0.105, Adjusted *R*^2^ = 0.086						
**Model 2**							
Age	0.313	0.079	0.162	3.947	0.000	0.157	0.469
Gender	–0.657	0.230	–0.114	–2.852	0.005	–1.109	–0.204
Mother’s ed.	–0.020	0.155	–0.007	–0.129	0.897	–0.323	0.284
Father’s ed.	0.380	0.168	0.117	2.269	0.024	0.051	0.710
Sibling no.	0.429	0.165	0.130	2.595	0.010	0.104	0.753
Birth order	–0.169	0.183	–0.047	–0.919	0.359	–0.529	0.192
Monthly inc.	–0.110	0.130	–0.040	–0.842	0.400	–0.365	0.146
Father’s emp.	0.118	0.588	0.008	0.200	0.842	–1.037	1.272
Mother’s emp.	–.336	0.255	–0.058	–1.318	0.188	–0.837	0.165
Academic ach.	0.362	0.234	0.066	1.546	0.123	–0.098	0.822
Social rel.	–0.438	0.240	–0.075	–1.823	0.069	–0.910	0.034
Daily tv. etc. usage	0.277	0.144	0.080	1.928	0.054	–0.005	0.560
Mindfulness	–0.116	0.019	–0.264	–6.254	0.000	–0.152	–0.080
Model statistics	*F* = 8.412, *p* = 0.000*, *R*^2^ = 0.163, Adjusted *R*^2^ = 0.144, *R*^2^ change = 0.058, DW = 1.337						

β, Regression Coefficient; DW, Durbin Watson; CI, Confidence Interval.

*p < 0.05.

The regression coefficient was also significant (*t* = –7.221, *p* < 0.001), indicating that for each one-unit increase in mindfulness, there was an associated 0.127-unit decrease in online risk-taking (β = –0.127). It should be noted that mindfulness accounted for only approximately 9% of the variance in online risk-taking behaviors (Adjusted *R*^2^ = 0.082), indicating a modest effect size and suggesting that many other factors also contribute to children’s online risk-taking.

In addition to simple linear regression analysis, in order to to assess the unique effect of mindfulness on online risk-taking behavior, a hierarchical multiple regression analysis was conducted. In the first step (Model 1), demographic and behavioral variables such as age, gender, parental education, number of siblings, birth order, monthly income, parental employment status, academic achievement, social relationships, and daily screen time were entered into the model. This initial model significantly predicted online risk-taking scores (*F* = 5.482, *p* < 0.001), explaining 10.5% of the variance (*R*^2^ = 0.105, Adjusted *R*^2^ = 0.086). In the second step (Model 2), mindfulness was added to the model to determine its unique contribution. The inclusion of mindfulness significantly improved the model fit (*F* = 8.412, *p* < 0.001), increasing the explained variance to 16.3% (*R*^2^ = 0.163, Adjusted *R*^2^ = 0.144), with an *R*^2^ change of .058. Importantly, mindfulness emerged as a significant negative predictor of online risk-taking behavior (β = –0.116, Std. β = –0.264, *t* = –6.254, *p* < 0.001), indicating that higher levels of mindfulness are associated with lower engagement in online risk-taking, even after controlling for all other variables in the model. This finding underscores the unique and substantial role of mindfulness in predicting online risk-taking tendencies.

## 4 Discussion

This study provides important information about the interaction between online risk-taking, mindfulness, and demographic-personal characteristics among middle school-aged children living in the Çankaya district of Ankara, Türkiye. A series of statistical analyses was conducted to examine these relationships in depth. Given that mindfulness is increasingly recognized as a protective factor against various behavioral risks, the findings of this study highlight the need for interventions aimed at increasing mindfulness and reducing online risk-taking behaviors among this vulnerable population group. Such interventions can be structured by considering the relationship between certain demographic and personal variables and conscious mindfulness and online risk-taking. This research focuses on middle school-aged children who are increasingly exposed to digital environments and are therefore more susceptible to online threats. To our knowledge, this study is one of the first to comprehensively examine the relationship between online risk-taking and mindfulness with demographic and personal characteristics among children in this age group in the Turkish context.

### 4.1 Mindfulness is a protective factor against online risk-taking

The findings of this study reveal a significant inverse relationship between online risk-taking and mindfulness among middle school-age children. Specifically, higher levels of mindfulness were associated with lower engagement in online risk-taking behaviors, while lower mindfulness levels corresponded with increased online risk-taking. These results highlight the potential of mindfulness as a protective factor capable of mitigating harmful digital behaviors in children. They further suggest that mindfulness-enhancing interventions could serve as an effective strategy for reducing children’s vulnerability to online risks. This conclusion aligns with existing literature. For instance, mindfulness-based training programs have been shown to reduce problematic online gaming while concurrently enhancing mindfulness in adolescents (Bekir, 2024). Theoretical prevention frameworks have also demonstrated efficacy in decreasing stress-related problematic gaming (Yuan and Liu, 2021). Moreover, parental mindfulness has been positively associated with reductions in children’s behavioral problems (Işık, 2020). Conversely, low mindfulness levels have been linked to increased incidences of cyberbullying (Kozan et al., 2018; Yuan and Liu, 2021; Yuan et al., 2020), high mindfulness of low-level gaming addiction (Shabban, 2023); and individuals with internet addiction often exhibit significantly lower mindfulness compared to non-addicted counterparts (Zgierska et al., 2008). Our findings in this study are consistent with previous findings and demonstrate a negative association between mindfulness and online risk-taking in children. These results emphasize the importance of developing and implementing mindfulness-based programs designed to meet the developmental needs of middle school-aged children, thereby demonstrating that the likelihood of children engaging in online risk-taking behavior can be significantly reduced.

### 4.2 Demographic—personal variables and online risk-taking

Our findings indicate that middle school-age children aged 13–18 exhibit significantly higher levels of online risk-taking compared to their younger counterparts aged 10–12. This trend suggests that children may be more prone to engaging in risky online behaviors as they grow older due to increased autonomy, greater access to digital technologies, and greater participation in online social interactions. These findings are consistent with previous research, which has demonstrated that older adolescents encounter more communication-related online risks (Livingstone et al., 2018); older teenagers engage in more online communication activities than younger children and therefore face more communication risks (Livingstone and Helsper, 2007); and that variables such as age and gender substantially influence children’s internet usage patterns (Holloway et al., 2013). From a developmental perspective, adolescence is marked by heightened vulnerability to risk-taking behaviors, attributed to ongoing brain maturation, psychosocial changes, and insufficient self-regulation capacities (Akyel et al., 2018; Steinberg, 2004; Steinberg, 2004). Additionally, the propensity for novelty- and sensation-seeking is more pronounced during this period, further fueling engagement in high-risk activities (Irmak et al., 2008). A substantial body of literature supports the assertion that risk-taking behaviors escalate with age (Aras et al., 2007; Crone and Dahl, 2012; Eisen et al., 2000; Hale et al., 2014; He et al., 2004; IOM and NRC, 2011; Jackson et al., 2012). In line with these findings, our study suggests that adolescence is a critical period during which online risk-taking behavior intensifies, and age may emerge as a key determinant of increased susceptibility to digital threats.

Other findings show that boys score significantly higher on online risk-taking than girls (*p* = 0.033, *d* = 0.179). This result is consistent with previous research suggesting that male adolescents tend to engage in risky online behaviors. Studies show that boys are more likely than girls to share personal information, communicate with strangers, or access age-inappropriate content (Livingstone and Helsper, 2007; Šmahel and Blinka, 2012). The small effect size observed here (Cohen’s *d* = 0.179), although statistically significant, suggests a modest gender difference and a significant overlap between boys’ and girls’ behavior. This result may reflect similar usage in online environments, with girls being as active online as boys (Lenhart et al., 2010).

This finding suggests the need for gender and age-sensitive interventions that consider different patterns of online behavior. Future research could utilize longitudinal designs to examine the relevance of age and gender differences in technology use by examining underlying motivational and contextual factors.

Other findings show that children with good academic achievement show lower online risk-taking (*p* = 0.012). This result suggests that academic success in children may be linked to safer online behavior. Previous studies suggest that academically successful children generally exhibit stronger self-regulation and executive functions, which may contribute to them making more informed and safer choices online (Duckworth et al., 2010; Zimmerman, 2000). Academic success is often associated with mindfulness and planning skills, which may predict less engagement in risky behaviors (Tangney et al., 2018). However, while the results are statistically significant, causality cannot be inferred; children who are naturally risk-averse online may have more time and resources to devote to schoolwork. Future research could utilize longitudinal designs or mediational analysis methods to determine whether other factors mediate this relationship. Understanding the dynamics between these factors could inform interventions that integrate safe online use with academic support for children.

Our study also found that school-age children who reported using televisions, tablets, phones, or computers for two or more hours per day exhibited significantly higher levels of online risk-taking compared to their peers with daily screen times of 0–2 h. This finding aligns with previous research demonstrating that excessive exposure to high-risk television programming (Potts et al., 1994), violent media content (Krcmar and Greene, 2000), and mobile phone overuse or addiction (Chee et al., 2021; De-Sola et al., 2017; Tang et al., 2017; Wang et al., 2018) is positively associated with increased risk-taking behaviors among adolescents. The highly stimulating and rewarding nature of mobile phone use—offering constant novelty, immediate feedback, and social engagement—may intensify adolescents’ proclivity for risk, particularly in digital contexts (Lee et al., 2017; Lee et al., 2014). In this light, our findings reinforce the growing body of literature suggesting that prolonged screen exposure serves as a risk factor for online behavioral vulnerabilities. These results underscore the critical importance of monitoring and regulating children’s digital media consumption as a strategic intervention to reduce their susceptibility to online risk-taking behaviors.

### 4.3 Demographic—personal variables and mindfulness

One notable finding of this study was that middle school-age children aged 13–18 exhibited significantly lower levels of mindfulness compared to their younger peers in the 10–12 age group. This result suggests that mindfulness may decrease during adolescence. Interestingly, this result contrasts with previous findings, such as those by Karadoğan (2022), which reported that age did not significantly predict mindfulness levels (Karadoğan, 2022). A possible explanation for our finding lies in the cognitive and emotional differences between children and adolescents. Younger children may be more receptive to the benefits of mindfulness interventions, as they are still developing foundational cognitive and emotional regulation skills, whereas adolescents often face more complex psychosocial challenges that may interfere with the adoption or efficacy of mindfulness practices (Dunning et al., 2019). Given that mindfulness research in childhood and adolescence remains a relatively nascent field, future studies should further explore how age-related developmental factors influence mindfulness capacities and intervention outcomes. Such research is essential to inform age-appropriate mindfulness-based strategies that effectively support mental and behavioral wellbeing across developmental stages.

Another finding of our study also indicated that boys exhibited higher mindfulness levels than girls. This gender-based difference contributes to the ongoing discourse in the literature, which presents mixed results regarding mindfulness and gender. Several studies have reported no significant difference between male and female adolescents in overall mindfulness levels (Bellinger et al., 2015; Brown and Ryan, 2003; Çolak, 2024; Düşünceli and Demirel, 2023; Güldal, 2019; Yıldırım and Tanrıverdi, 2024; Zeidan et al., 2018). However, some research suggests that gender differences may emerge at the subscale level: for instance, male adolescents have been found to score higher in non-reactivity, non-judgment, and self-acceptance, whereas females tend to score higher in attention and mindfulness (Kısmetoğlu, 2019). Further complicating the picture, other studies have yielded conflicting conclusions, with some reporting higher mindfulness scores among females (Şahin, 2019) and others showing significantly greater mindfulness among males (Altan, 2019). Contrary to expectations, mindfulness was found to be higher among boys than girls, despite many studies reporting no significant gender differences or higher levels among girls. This inconsistency may be due to cultural differences in gender socialization or to differences in how mindfulness is expressed and measured across contexts. These inconsistencies may be attributed to variations in sample characteristics, cultural contexts, or the measurement tools employed. Taken together, these findings suggest that gender may influence specific dimensions of mindfulness, but additional research is needed to clarify these relationships and to understand the underlying mechanisms driving them.

Another noteworthy finding of our study was that first-born children exhibited significantly higher levels of mindfulness compared to their second-born counterparts. This result is consistent with existing literature that underscores the influence of birth order on personality development. First-born children are frequently characterized as more compliant, rule-oriented, and assertive, traits often attributed to their distinct relational dynamics with parents (Adler, 2014; Forer, 2013; Paulhus et al., 1999). Further supporting this notion, prior research has demonstrated that certain dimensions of mindfulness tend to increase with age (Alispahic and Hasanbegovic-Anic, 2017; Güldal, 2019; Hohaus and Spark, 2013). Additionally, later-born children may receive comparatively less individualized parental attention and cognitive scaffolding, which could negatively impact their developmental trajectory in domains relevant to mindfulness, such as emotional regulation and executive functioning (Booth and Kee, 2009). Interestingly, while first-born children are often described as high-achieving, they are also more prone to attention-related challenges, a domain where mindfulness has demonstrated therapeutic benefits (Evrensel et al., 2015). Taken together, these findings suggest that the elevated mindfulness levels observed among first-born children may stem from both enhanced parental investment and their greater exposure to structured developmental experiences early in life. Being the first child in a family can lead to greater responsibility, which in turn can lead to higher mindfulness.

Another finding from the study was that mindfulness was higher in children whose fathers did not work (*p* = 0.026, *d* = –0.375). This result offers interesting insight into family dynamics and children’s mindfulness development. The negative effect size (*d* = –0.375) indicates that children with unemployed fathers have moderately higher mindfulness compared to children with employed fathers. This result may be because unemployed fathers have more time to participate in practices and activities that enhance children’s mindfulness (Cabrera et al., 2018; Santis and Barham, 2017). An alternative explanation for the higher mindfulness scores in children of unemployed fathers may relate to socioeconomic pressures that foster early sense of responsibility in children, rather than to fathers’ availability. This points to a complex set of contextual influences that cannot be fully captured by quantitative measures. Future research should examine not only paternal employment status but also the nature and type of paternal involvement to better understand the mechanisms behind this finding. Longitudinal studies and studies that consider cultural differences could clarify whether this pattern is generalizable across different populations.

Another finding of our study revealed that middle school-age children with good academic achievement demonstrated significantly higher mindfulness compared to their peers with moderate or low academic performance (*p* = 0.000). This result shows that academic success in children may be linked to more conscious attitudes. This finding is consistent with a growing body of literature suggesting a strong positive association between mindfulness and academic success. For instance, mindfulness has been linked to higher grade point averages among high school students; positively correlated with overall school achievement; and shown to predict mathematics performance in children (Güldal, 2019; Kocabal and Ceylan, 2022; Zuo and Wang, 2023). These findings reinforce the view that academic achievement may serve as a robust predictor of mindfulness in middle school-age populations. One possible explanation is that students with higher levels of mindfulness are better equipped to manage cognitive and emotional distractions, thereby enhancing their capacity to sustain attention and engage deeply in learning tasks. Furthermore, mindfulness may bolster students’ intrinsic motivation and self-regulatory abilities, allowing them to recognize personal strengths, adjust learning strategies accordingly, and maintain greater perseverance in the face of academic challenges.

Other findings demonstrated that middle school-age children with good social relationships exhibited significantly higher mindfulness compared to their peers with medium/poor peer relationships (*p* = 0.000, *d* = 0.665). This result shows that positive peer relationships can strongly support mindfulness in children. This observation aligns with existing research indicating that higher mindfulness levels are positively associated with enhanced character strengths, including perseverance, social intelligence, gratitude, and hope (Güldal, 2019). Moreover, mindfulness has been shown to improve interpersonal communication in young adults; reduce social anxiety in adolescents, and negatively correlate with general social anxiety among university students (Çolak, 2024; Dinç, 2019; Durusoy, 2019). These findings collectively suggest that mindfulness fosters social and emotional competencies by enhancing an individual’s ability to remain present, attuned, and responsive in social interactions. By promoting greater emotional awareness, intentional communication, and self-regulation, mindfulness may enable children to navigate social situations with increased empathy and tolerance. Consequently, cultivating mindfulness in middle school-age children may serve as a valuable approach to strengthening their social relationships and overall social functioning.

Another noteworthy finding of this study was that middle school-age children who reported screen use (including television, tablets, smartphones, or computers) of 0–2 h per day had significantly higher mindfulness compared to those using screens for more than 2 h daily (*p* = 0.001). This pattern is consistent with previous research indicating a negative association between mindfulness and excessive technology use. For example, studies have shown that mindfulness is inversely related to smartphone addiction; prolonged social media engagement beyond 1 h per day; digital game addiction, and internet addiction (Cırcır and Bayar, 2023; Çay, 2019; Düşünceli and Demirel, 2023; Keskin, 2019; Şehidoğlu, 2014). However, contrasting findings also exist, with some studies reporting no significant relationship between mindfulness and variables such as general internet access, digital game use, or average daily social media activity (Çolak, 2024; Karzı, 2020). Despite such inconsistencies, the current results support the broader hypothesis that prolonged daily screen exposure may be detrimental to mindfulness. Children with higher mindfulness levels may possess greater self-regulatory abilities, enabling them to identify and resist habitual or emotionally driven screen use. Such individuals are more likely to remain attuned to their internal states and external environments, thereby reducing dependency on digital stimuli. Thus, mindfulness may function as a psychological buffer, helping prevent the development of problematic screen-related behaviors, including digital and gaming addictions.

## 5 Conclusion

In conclusion, this study underscores the critical role of mindfulness in mitigating online risk-taking behaviors among middle school-age children. The findings demonstrated a significant negative correlation between middle school-age children’s mindfulness levels and their engagement in online risk-taking behaviors. Specifically, higher mindfulness was associated with reduced propensity for risky online conduct. These results suggest that mindfulness may play a protective role by enhancing children’s capacity to recognize and evaluate potential online threats, thereby promoting safer digital behavior. Mindfulness is a significant and inverse predictor of online risk-taking. Children and adolescents with higher levels of mindfulness exhibit less online risk-taking. Although mindfulness alone only explains a limited portion of the variance (8.4%) in the current study, it is seen as an important protective factor in reducing online risks.

This study also examined the relationships between online risk-taking, mindfulness, and various demographic and personal characteristics in a sample of middle school-aged children. Specifically, it explored the predictive capacity of mindfulness and selected individual variables—such as age, gender, birth order, academic performance, social relationships, and screen time—on children’s propensity to engage in risky online behaviors. By identifying key factors that influence both mindfulness and online risk-taking, the research provides valuable insights for the development of targeted interventions aimed at enhancing children’s digital safety and psychological resilience.

According to the results obtained from the research, it was seen that children with low mindfulness levels may be more likely to engage in online risk-taking behaviors in online environments. Notably, this vulnerability was especially evident in specific subgroups, including adolescents aged 13–18, boys, those with lower academic performance, and those who spend more than 2 h a day in front of the screen.

These findings highlight the urgent need for targeted mindfulness-based interventions and educational initiatives aimed at these high-risk groups. Programs that foster mindfulness can equip children with skills to better understand and regulate their thoughts and emotions, enhance attentional control, and ultimately make safer, more informed choices when navigating online spaces. By addressing these cognitive and emotional dimensions, such initiatives may serve as effective tools in reducing online risk exposure and promoting safer digital engagement among children. Among the variables analyzed, age, gender, academic achievement, and daily screen time emerged as significant predictors of online risk-taking. Specifically, online risk-taking behaviors were found to increase with age, suggesting a developmental trajectory associated with greater autonomy, greater internet access, and greater exposure to peer influence. The reason why boys’ online risk-taking levels are slightly higher than girls’ may be due to their tendency to seek more excitement and their greater likelihood of exploring unsupervised online spaces. Children with higher academic achievement may tend to take fewer online risks, have better self-regulation skills, and have more structured routines that limit unsafe online behavior. The reason why increased daily screen time use is linked to greater online risk-taking may be that greater online exposure and increased time online increase the likelihood of encountering or participating in risky activities. All these results underline the importance of digital media regulations among the young population.

The study also identified several demographic and personal characteristics that significantly influenced mindfulness levels in middle school-age children. These included age, gender, birth order, father’s employment status, academic achievement, social relationships, and daily screen time. The findings revealed that mindfulness levels tended to decline with increasing age. This may be due to increased cognitive, social, and emotional demands during adolescence and greater exposure to distracting digital environments that challenge attention and self-regulation. Male participants demonstrated significantly higher mindfulness levels compared to females. This difference may stem from gender-related variations in coping styles and emotional regulation. Additionally, first-born children exhibited greater mindfulness than their second-born counterparts. Firstborn children may have developed greater mindfulness due to their parental attention and responsibilities that encouraged self-regulation. The reason behind the higher level of mindfulness in children whose fathers do not work is that when fathers are more present at home, children may have received additional guidance and support that fosters conscious attitudes and thoughtful habits. The reason for the increased mindfulness level of children with high academic success may be that high achievers have greater self-discipline and attention-gathering skills. While children who reported strong, positive relationships with peers also demonstrated increased mindfulness levels. This may be because feeling emotionally safe, empathy, and supportive social environments encourage mindfulness by providing opportunities to practice self-awareness. The reason why children with less daily screen time were found to have higher levels of awareness may be that limiting screen time reduces distraction, mental overload, and moderate screen use provides more space to focus on the moment.

These findings carry important implications for parents, educators, and mental health professionals who support the development and wellbeing of children. Understanding the factors that influence online risk-taking, and mindfulness can inform the design of preventive strategies, educational curricula, and therapeutic interventions aimed at promoting safer online behaviors and improving mindfulness in middle school-aged children. All these findings suggest that more research, more differently designed methods, school-based interventions, and more longitudinal studies are needed to determine whether increasing mindfulness in children is effective in reducing online risk-taking behavior.

When interpreting the findings of this article, it is important to bear in mind that there are certain limitations. Firstly, the cross-sectional nature of the design prevents causal inferences from being made about the relationship between mindfulness and online risk-taking. For this reason, it is useful to interpret the findings as correlations rather than predictions. Secondly, since the data is based on measurements of how children express themselves, it may be subject to biased responses such as social desirability and false recall. Although we used measurement tools with proven validity and reliability to eliminate these limitations, the use of a single source of information may have affected the results. Thirdly, although we used a robust random sampling strategy to increase representativeness, the fact that all participants live in the Çankaya district of Ankara, Türkiye, may affect the generalizability of the findings due to the sample having specific geographical, demographic, and personal characteristics. Finally, the fact that mindfulness explains only approximately 12% of the variance in online risk-taking behavior in the regression model (adjusted *R*^2^ = 0.117) suggests that other factors may also influence these behaviors.

Future researchers can use mixed method designs to gain a more comprehensive understanding of online risk-taking behavior and assess a wider range of risk and protective factors. Despite all these limitations, this study makes an important contribution to the field by identifying some demographic and personal characteristics of mindfulness and online risk-taking behavior among middle school children.

## 6 Future directions and recommendations

While this study offers valuable contributions to the field, it also highlights several directions for future research and practice. Given the limitations noted above, future research could utilize longitudinal and mixed method designs to better understand the causal mechanisms between mindfulness and online risk-taking. Additionally, variables such as parenting styles, school environment, and peer dynamics could help provide a more nuanced model of children’s online risky behavior.

From a practical perspective, the findings in this study highlight the potential of mindfulness-based programs to reduce online risk-taking. Such interventions can be integrated into school settings and digital literacy curricula, particularly for the high-risk groups identified in this study: older adolescents, boys, and children who spend more time in front of screens. By tailoring interventions to children’s developmental needs and contextual realities, educators, parents, and policymakers can create stronger frameworks for digital health and wellbeing.

Outside of schools, parents play a critical role in guiding children’s online activities by modeling responsible technology use, communicating openly with their children, and setting appropriate boundaries. Mental health professionals can also incorporate mindfulness-based practices into preventive and therapeutic programs targeting young people’s digital behavior. Furthermore, collaboration with policymakers and child protection organizations is essential to develop evidence-based strategies and regulations that protect children online. Technology companies and internet service providers should also be involved in creating safer online environments by adopting child-friendly security measures and supporting digital literacy initiatives.

In summary, this research provides a foundation for both theoretical and applied developments. Future research, combining further empirical research with school- and community-based awareness practices, can help protect children in digital environments. By involving multiple stakeholders, including families, schools, health professionals, policymakers, and technology developers, prevention efforts can be made more sustainable and effective.

## Data Availability

The datasets generated and/or analyzed during the current study are not publicly available due to ethical restrictions involving minor participants but are available from the corresponding author upon reasonable request after anonymization.
